# Factors associated with health-related quality of life among Chinese caregivers of the older adults living in the community: a cross-sectional study

**DOI:** 10.1186/1477-7525-10-143

**Published:** 2012-11-27

**Authors:** Xiaoshi Yang, Yiming Hao, Shernelle Marlah George, Lie Wang

**Affiliations:** 1 Department of Social Medicine, School of public health, China medical university, No.92 North second road, Heping District, Shenyang, 110001, China

**Keywords:** Older adults, Subjective caregiver burden, Sense of coherence, HRQOL

## Abstract

**Background:**

Under the culture of filial piety and due to the Confucianism spirit in China, family caregivers usually undertake the responsibilities of caring for the older adults. They usually suffer from a heavy burden which is believed to impair their mental and physical health. Thus this study aims to describe the health-related quality of life (HRQOL) among Chinese caregivers of the older adults living in the community and explore the predictors of caregivers’ HRQOL.

**Methods:**

A cross-sectional study was conducted through convenience sampling. The study population was composed of 1,144 caregivers of older adults who suffered from one or more types of chronic diseases in 15 communities in 3 eastern cities of China. Family caregivers were interviewed face-to-face using the 36-item Short-Form Health Survey (SF-36) and the ZARIT Caregiver Burden interview (ZBI) scales. The Antonovsky's Sense of coherence (SOC) scale was also used to measure personal coping capability of the caregivers. Hierarchical multiple regression analysis (HMR) was performed to explore the predictors of caregivers’ HRQOL.

**Results:**

The majority of the caregivers were females (60.0%) or adult children (66.5%). Mental QOL was significantly lower than physical QOL. Hierarchical multiple regression analysis showed that Demographic Characteristics of Caregivers, Patients’ Characteristics, and Subjective Caregiver Burden explained most of the total variance of all aspects of HRQOL. While, Objective Caregiving Tasks was only associated with physical QOL. Subjective Caregiver Burden was the strongest predictor of both physical and mental QOL. SOC was also a strong predictor of physical and mental QOL.

**Conclusions:**

The mental QOL of the caregivers of older adults was disrupted more seriously than physical QOL. Additionally, Subjective Caregiver Burden might decrease caregiver’ health. A decrease in caregiver burden could promote better management of caregiving tasks, and improve HRQOL. Also, coping capabilities, like SOC, are needed to decrease the impact of caregiving on HRQOL of Chinese caregivers of the older adults.

## Background

In recent times, China’s concern about the national burden placed on the country by the elderly has heightened. The population of the elderly in China has been aging dramatically, and the number of Chinese people aged 60 and over will increase to 400 million by the year 2050, accounting for 25% of the whole population [1].

Advances in healthcare and nutrition, combined with the one child policy, have led to a small family size, the increase of empty-nest families as well as elderly people living alone. These have caused a corresponding rise of the elderly dependency ratio. The result is a population bulge called the "4-2-1" phenomenon, in which one child is supporting two parents and four grandparents. Older adults are at a high risk for chronic diseases. Once the disease occurs, the high cost of care is overwhelming for many families, which could result in great financial and caregiving burden. Owing to the paucity of formal institutional methods for long-term care, and due to the culture of filial piety and the Confucianism spirit in China, family members are usually left with the responsibilities of caring for the elderly.

Caring for the elderly not only affects caregivers’ daily lives, but also poses dramatic and considerable psychological and physical challenges. The negative effects of such strain affect their present and future daily life and health. Caregiving requires much time and effort, great material and emotional expenditure, and also hinders caregivers’ social engagements. The role of caregiving can be highly stressful, burdensome, and hence, compromises the caregiver’s quality of life, which could lead to considerable physical, psychological and social impairments [2]. Research has consistently reported that increased caregiver burden was associated with a decline of well-being and quality of life in many countries [3,4]. Schulz found that caregivers who were providing care and experiencing caregiver burden and strain had a 63% higher risk of mortality than non-caregivers [5]. This, in more cases than expected, exceeds that of the elderly themselves. Numerous studies have also shown that caregivers have a higher risk of both physical and mental health problems, as compared to non-caregivers [6-8]. Caregivers are usually the hidden victim of the disease, and so have been labeled the “hidden patients” [9].

Sense of Coherence (SOC) during the process of caregiving has yielded various findings as to caregivers’ health. Sense of coherence is the ability of caregivers to mobilize their coping resources during periods of caregiving [10,11]. Antonovsky [12] found that people with a stronger SOC despite extremely pressing circumstances such as providing caregiving to their family members could positively deal with stress and protect health. Caregivers with a strong SOC, which refers to their ability to respond to stressors by the appropriate use of adaptive coping resources, can avoid breakdown, alleviate mental health problems and promote physical health.

The effects of caring for older adults on caregiver’s health have moved to the forefront nowadays in Western countries, Japan and Taiwan. However, very little is known about caregivers of older adults in mainland China. Thus, this study aims to describe the quality of life (QOL) among Chinese caregivers of older adults and explore the predictors of caregivers’ QOL. Information derived from this study may provide evidence for policymakers to attenuate caregiver burden, promote quality of life and well-being among caregivers.

## Methods

### Study design and sample

A cross-sectional study was conducted from November 2010 to August 2011. The sample used in this study consisted of 1,500 caregivers of the older adults living in the community. A convenience sample of primary family caregivers of the elderly with one or more types of chronic diseases was selected from 15 communities in 3 eastern cities of China (Shenyang of Liaoning Province, Yantai of Shangdong Province and Nanqiao of Shanghai). All cities contain a large percent of the elderly population, locating at the north, central and south of eastern China. Primary family caregivers of ages 18 years and older, who had cared for the elderly with the ages of 60 years and older, for at least the past 6 months, were enrolled in this study. This time frame was chosen based on the criteria for the period of long-term care for older adults [13,14].

Family doctors from the community service centres who were participating in the project identified the individuals who met the criteria and explained the study to them and their primary family caregivers. If the caregivers agreed to participate, they were asked to sign consent forms and enrolled in the study. All the participants were well-informed about the content and aim of the study. After obtaining the written informed consent about the conduct of the survey, caregivers were interviewed face-to-face by doctors or trained surveyors in the home of the elderly. The procedures followed were in accordance with and approved by, the ethical standards of the Committee on Human Experimentation of China Medical University.

Of the 1,500 caregivers identified, 210 declined to participate resulting in an 86.0% consent rate. Eighty-five were excluded because they dropped out of the study, and sixty-one were excluded because of missing values exceeding 10% in the questionnaire. The characteristics (Sex, Race, and Objective Caregiving Loads) of the remaining 1,144 caregivers included in the analysis were similar to those of the 150 caregivers who were excluded.

### Caregivers' quality of life

#### Assessment of QOL

The 36-item Short-Form Health Survey (SF-36) [15,16] was applied to assess QOL of caregivers. The SF-36 consists of 36 items that measures eight different dimensions of health: physical function, role limitations related to physical problems, bodily pain, general health perception, vitality, social functioning, role limitations due to emotional problems and mental health. These dimensions can be categorized into physical component summary (PCS) and mental component summary (MCS). The health concepts are described by a range in score from 0 to 100, with higher scores indicating better health. The internal consistency of the questionnaire was Cronbach’s α = 0.81 in this study.

### Independent variables

#### Demographic Characteristics of Caregivers

Demographic Characteristics of Caregivers included age, gender, education level (<senior middle school/≥senior middle school), marital status (married/other), currently employed (yes/no), financial status, number of caregivers, and relationship to the patient. Family caregivers were asked whether they suffered from any chronic disease. ‘Chronic disease’ was defined as ‘yes’ if any common chronic disease (e.g., hypertension, cardiovascular disease, diabetes, and arthritis) had ever been diagnosed.

### Characteristics of the Elderly

These questionnaires were completed by the caregivers through structured face to face interviews conducted by the doctors and trained interviewers. The doctors and interviewers acquired information on gender, age, education level, marital status (married/other), income level, functional status, and number of chronic diseases. Functional status was measured by the Activities of Daily Living Scale (ADL) designed by Lawton and Brody in 1969 [17]. ADL, consisting of 14 items, measures the degree of independence in basic (e.g., dressing and grooming) and instrumental (e.g., medication and financial management) daily activities. Each item is scored on a 4-point scale, reflecting independence, the need for little assistance, the need for much assistance or dependence. Total scores range from 0 to 42, with higher scores reflecting higher levels of dependency.

### Objective Caregiving Loads

Objective Caregiving Loads measured in this study included care tasks, level of care, and the amount of time spent in providing care each day. Care tasks and level of care were measured by the amount of assistance provided in personal care, homemaking, transportation, and health care, as determined by Emanuel et al. [18]. Family caregivers were asked to rate the amount of assistance they provided to the patients in each of these four categories on a four-point scale ranging from “none at all” to“a lot”. Each item has four response categories: 0=none at all, 1=sometimes, 2=frequently and 3=always. A composite score for the total assistance provided was computed by summing the scores of those four items (Cronbach's alpha=0.91). The number of caregivers refers to the total number of the caregivers who provide care to the elderly including primary and secondary family caregivers. The **amount of time spent each day in caregiving** was classified into less than or equal to 4, 5–6, 7–8, and 9–24 hrs/day. Care duration was classified into less than or equal to1, 1–4 and more than 4 years.

### Subjective Caregiver Burden

Subjective Caregiver Burden was measured with The Zarit Caregiver Burden Interview (ZBI) [19], which was developed by Zarit in 1985. It is composed of 22 items graded on a scale from 0 to 4, according to the presence or intensity of an affirmative response. The questions refer to the caregiver/patient relationship and evaluate the caregiver’s physical health, psychological well-being, finances, and social life. Each item has five response categories: 0=none at all, 1=occasionally, 2=sometimes, 3=frequently and 4=always. Caregiver burden is evaluated by means of the total score obtained from the sum of the questions. The total score ranged from 0 to 88, with higher scores indicating a heavier caregiver burden. The Chinese version of ZBI had been established [20] in previous studies and had a higher reliability and validity. The reliability of this study was 0.85.

### Personal Coping Capability

The Chinese edition of the Antonovsky's Sense of coherence (SOC) scale [12] was used to measure personal coping capability. It consists of 13 items, including three critical attributes within the SOC construct: meaningfulness, comprehensibility, and manageability. Respondents were asked to rate themselves on a seven-point Likert scale. The total score ranged from 13 to 91, with higher scores indicating a stronger SOC. The Cronbach's alphas of the SOC scale from published studies ranged from 0.70–0.92 for various samples [21]. The Cronbach's alpha coefficient was 0.83 for SOC in this study.

### Statistical analysis

The distributions of QOL in categorical variables were evaluated using t-test and one-way analysis of variance (**ANOVA**). Correlations between QOL and continuous variables were examined by Pearson’s correlation. If the correlation between two variables was more than 0.5, these variables were regarded as co-line variables, and were adjusted in the multivariate analysis. In this study, no co-line variables were found. Hierarchical Multiple Regression (HMR) analysis was conducted to test the incremental variance by any given set of independent variables. The QOL scores were used as dependent variables. The independent variables were entered in the following steps: Step 1: characteristics of the elderly; Step 2: demographic characteristics of the caregivers; Step 3: objective caregiving loads; Step 4: subjective caregiver burden and; Step 5: sense of coherence. The analysis proceeded in stages by successively including several blocks of independent variables in the regression model. Blocks of variables entered in later stages were thus tested for their extra contribution after the contributions of earlier-entered variables had been removed. The relative importance of the variables retained in the final multiple regression models contributed to the explained variance of the QOL, which was represented as the standardized β [22]. Fit of the model was assessed using the R^2^-value. Statistical analysis was performed using the Statistical Package for Social Science Version 11.5. A two-tailed probability value of less than 0.05 was considered to indicate statistical significance.

## Result

### Description of the characteristics of the caregivers and the elderly

The 1144 caregivers who completed the questionnaires effectively constituted a valid response rate of 76.3%. The basic characteristics of the participants are provided in Table 1. The average age of the older adults was 72.1±8.7 years old. There were 630 (55.1%) males and 514 (44.9%) females. The older adults’ chronic diseases categories in this study were stroke (184), arthritis (246), cervical spondylosis (157), hypertension (498), coronary heart disease (243), diabetes (240) and cancer (67). The older adults with an educational level of higher than junior middle school accounted for more than 52.4%. Approximately 57.0% of the older adults are married, and their mean ADL scale was 26.2, SD 12.7.

**Table 1:** Demographic characteristics of caregivers and the older adults

**Variables**	**N**	**%**	**Variables**	**N**	**%**
**Caregivers’ demographic characteristics**			**The elderly’ characteristics**		
**Gender**			**Gender**		
Male	460	40.21	Male	630	55.07
Female	684	59.79	Female	514	44.93
**Age (years old)**			**Age (years old)**		
≤40	444	38.81	60-69	453	39.6
41-49	391	34.18	70-79	438	38.3
≥50	309	27.01	≥80	253	22.12
**Marital status**			**Marital status**		
Married	995	86.98	Married	652	56.99
Others	149	13.02	Others	492	43.01
**Educational level**			**Educational level**		
<Senior middle school	512	44.76	<Junior middle school	545	47.64
≥Senior middle school	632	55.24	≥Junior middle school	599	52.36
**Monthly household income of caregivers(Yuan)**			**Income level of the elderly(Yuan)**		
<1000	160	13.99	<1000元	342	29.90
1000-1999	291	25.44	1000-1999	305	26.66
2000-2999	339	29.63	2000-2999	315	27.53
≥3000	352	30.77	≥3000	182	15.91
**Chronic diseases**			**Number of chronic diseases**		
No	413	36.10	1.00	638	55.77
Yes	731	63.90	2.00	281	24.56
**Living with the elderly**			≥3.00	225	19.67
Yes	534	46.68	**Caregiving in assistance for daily living**		
No	561	49.04	Not at all	215	18.79
Missing	49	4.28	Occasionally	537	46.94
**Relationship to the patient**			Frequently	244	21.33
Spouse	111	9.70	Always	148	12.94
Daughter	330	28.85	**Caregiving in assistance for housekeeping**		
Son	318	27.80	Not at all	138	12.06
Daughter in law	113	9.88	Occasionally	501	43.79
Others	272	23.78	Frequently	311	27.19
**Numbers of caregivers**			Always	194	16.96
1	215	18.79	**Caregiving in assistance for transportation**		
2	369	32.26	Not at all	180	15.73
3	249	21.77	Occasionally	481	42.05
≥4	264	23.08	Frequently	264	23.08
Missing	47	4.11	Always	204	17.83
**Currently employed**			Not at all	15	1.31
no	330	28.85	**Caregiving in assistance for health care**		
yes	788	68.88	Not at all	188	16.43
Missing	26	2.27	Occasionally	502	43.88
**Time spent in caregiving everyday**			Frequently	282	24.65
<4h	308	26.92	Always	172	15.03
5-6h	391	34.18			
7-8h	196	17.13			
9-24h	249	21.77			
**Care duration (years)**					
≤1	317	27.71			
1-4	417	36.45			
≥4	410	35.84			

Caregivers’ average age was 44.3±11.5 years old, ranging from 20–79 years old. They consisted of 460 (40.0%) males and 684 (60.0%) females. In these subjects, the majority of the caregivers were adult children (66.5%). The percentage of currently employed caregivers was 68.9%. Nearly half of the family caregivers sometimes provided assistance to the elderly in personal care, homemaking, transportation, and health. The median of care duration for patient was 2.5 years, ranging from half a year to 32 years. More than one third (34.2%) of the caregivers spent 5–6 hours in caregiving each day. The mean ZBI scale was 27.3, SD 15.0. However, caregivers reported that caregiving created substantial positive effects like SOC in caregiving. The strength of SOC (56.1±5.6) in caregivers was moderately strong, which was equal to the level of SOC in cancer caregivers in Taiwan (59.8±15.9) [23].

### Description of the caregivers' QOL

In this study, the caregivers of older adults suffered lower levels of PCS and MCS, which was much lower than the levels of general mainland Chinese adults and the Chinese caregivers of the elderly in Hong Kong [24,25]; but higher than the caregivers of stroke patients [26]. The mean scores of caregivers’ QOL based on the demographic characteristics of caregivers and objective caring tasks are listed in Table 2. Mental QOL was significantly lower than psychical QOL. Female caregivers exerted a significantly lower level of MCS than the male caregivers. Caregivers with an education level of senior middle school or above displayed a higher degree of both PCS and MCS, while caregivers suffering from chronic disease tended to reported severer physical and mental QOL. Caregivers with lower monthly household income tended to exerted lower PCS and MCS. Spousal caregivers suffered lower levels of physical and mental QOL than those of other familial relationships to the older adult. Caregivers providing care to an ill family member, who suffered from more than one type of chronic disease, had QOL scores lower than those caring for the elderly with one chronic disease. Differences in age of caregivers, whether or not they were living together with the elderly, as well as care duration were not statistically significant. Family caregivers who cared for the elderly with higher levels of dependency (ADL scores), experienced a significantly lower level of physical and mental QOL.

**Table 2:** QOL Scores in characteristics of the older adults and their caregivers and objective caring loads

	**PCS**	**MCS**
**Caregivers’ demographic characteristics**		
**Gender**		
Male	74.09 ± 17.10	65.59 ± 15.98
Female	72.27 ± 17.44	63.35 ± 17.74*
**Age (years old)**		
≤ 40	74.17 ± 16.12	63.85 ± 16.46
41-49	72.52 ± 15.88	63.54 ± 16.01
≥ 50	71.89 ± 20.47	65.83 ± 19.17
**Marital status**		
Married	72.87 ± 17.46	64.51 ± 17.19
Others	73.87 ± 16.41	62.61 ± 16.22
**Educational level**		
< Senior middle school	69.82 ± 18.04**	62.41 ± 16.92**
≥ Senior middle school	75.61 ± 16.22	65.62 ± 17.04
**Monthly household income of caregivers(Yuan)**		
< 1000	65.70 ± 18.29**	58.67 ± 17.41**
1000-1999	72.40 ± 18.98*	64.13 ± 17.41
2000-2999	73.13 ± 16.95*	63.96 ± 16.84
≥ 3000	76.67 ± 14.57	67.12 ± 16.25
**Chronic diseases**		
No	76.12 ± 16.69**	65.84 ± 16.61**
Yes	71.25 ± 17.43	63.36 ± 17.28
**Living with the elderly**		
Yes	72.51 ± 17.50	63.35 ± 17.38
No	73.00 ± 17.32	64.70 ± 16.78
**Relationship to the patient**		
Spouse	68.24 ± 20.65**	62.38 ± 18.14
Daughter	72.50 ± 17.12	63.72 ± 18.21
Son	75.70 ± 15.75	65.99 ± 16.01
Daughter in law	73.33 ± 15.64	65.26 ± 15.77
Others	71.87 ± 18.47	63.33 ± 17.01
**Caregiving in assistance for daily living**		
Not at all	78.31 ± 14.07	69.31 ± 16.97
Occasionally	74.82 ± 16.18*	65.73 ± 16.27**
Frequently	65.13 ± 18.70**	57.71 ± 16.54***
Always	72.09 ± 18.66*	62.51 ± 17.63*
**Caregiving in assistance for housekeeping**		
Not at all	77.83 ± 14.77	70.16 ± 16.65
Occasionally	76.31 ± 15.79	66.44 ± 16.35*
Frequently	67.23 ± 17.46**	60.24 ± 16.44**
Always	70.55 ± 19.40*	60.93 ± 18.11**
**Caregiving in assistance for transportation**		
Not at all	77.41 ± 15.84	69.30 ± 17.55
Occasionally	73.09 ± 16.52**	63.82 ± 15.89**
Frequently	71.59 ± 17.56**	63.68 ± 16.99**
Always	70.58 ± 19.34**	60.96 ± 18.41**
Not at all		
**Caregiving in assistance for health care**	79.53 ± 14.87	69.73 ± 17.80
Not at all	73.38 ± 16.07*	64.70 ± 15.99*
Occasionally	68.64 ± 18.28**	60.28 ± 16.65**
Frequently	71.73 ± 19.58**	62.81 ± 18.36**
**Numbers of caregivers**		
1	72.64 ± 18.88	65.96 ± 17.29
2	73.62 ± 16.34	64.08 ± 16.85
3	74.51 ± 16.52	65.09 ± 18.21
≥4	70.22 ± 18.04*	62.02 ± 15.83
**Currently employed**		
no	68.02 ± 19.13**	60.46 ± 18.03**
yes	75.31 ± 15.96	65.94 ± 16.32
**Time spent in caregiving everyday**		
<4h	75.72 ± 16.13	66.64 ± 16.29
5-6h	66.06 ± 17.01*	57.88 ± 16.22*
7-8h	68.97 ± 18.60*	60.78 ± 16.54*
9-24h	63.67 ± 19.28**	55.00 ± 20.33**
**Time spent in caregiving everyday**	73.67 ± 19.28*	65.00 ± 20.33*
**Care duration (years)**		
≤1	73.48 ± 16.64	65.14 ± 17.41
1-4	72.73 ± 16.75	63.31 ± 17.01
≥4	72.92 ± 18.38	64.52 ± 16.87
**The elderly’ characteristics**		
**Gender**		
Male	73.28 ± 17.19	63.87 ± 16.83
Female	72.66 ± 17.49	64.74 ± 17.37
**Age**		
60-69	74.93 ± 16.91	65.29 ± 17.54
70-79	72.14 ± 17.28*	63.44 ± 16.21
≥80	71.05 ± 17.83**	63.83 ± 17.64
**Marital status**		
Married	73.96 ± 17.47	65.37 ± 16.98
Others	71.71 ± 17.07*	62.77 ± 17.13*
**Educational level**		
<Junior middle school	72.35 ± 17.25	64.36 ± 16.90
≥Junior middle school	73.67 ± 17.32	64.22 ± 17.25
**Income level of the elderly(yuan)**		
<1000元	70.30 ± 17.69**	62.76 ± 17.48
1000-1999	73.98 ± 17.06*	64.06 ± 16.22
2000-2999	74.45 ± 17.10	65.55 ± 17.47
≥3000	74.05 ± 17.13	65.29 ± 16.99
**Number of chronic diseases**		
1.00	74.71 ± 16.89	65.80 ± 16.75
2.00	72.52 ± 16.50*	64.22 ± 17.01
≥3.00	68.93 ± 18.74**	60.04 ± 17.39**
**ADL**		
<26	77.39 ± 15.75	67.95 ± 16.60
≥26	69.73 ± 17.72**	61.46 ± 16.90**

*P < 0.05,

**P < 0.01.

### The predictors of caregivers' QOL

Tables 3 and 4 shows the final results of the hierarchical multiple regression models of caregivers’ QOL. The total of 25.5% and 28.2% of variance were explained by the final regression model in PCS and MCS respectively. Results from the R^2^ change indicated that Subjective Caregiver Burden contributed most to the variance of both PCS and MCS. It also indicated that the demographic characteristics of the caregivers was the second highest contributor to the variance of PCS and MCS. Each block of independent variables made a significant contribution to the variance of caregivers' physical QOL. With the exception of objective caregiving tasks (P values in all variables of objective caregiving tasks were higher than 0.05), every block of the independent variables made a significant contribution to the variance of MCS. Subjective Caregiver Burden was the strongest predictor of all aspects of QOL, whereas SOC was positively associated with PCS and MCS.

**Table 3:** Hierarchical multiple regression predicting the PCS scores

	**Estimate (B)**	**Standardized estimate (β)**	**t**	***P***	**95% CI of β**	**R**^**2**^	**△R**^**2**^
Constant	67.905		8.191	<0.001	51.636~84.174		
**Block 1. The Elderly’ Characteristics**						**0.034**	**0.034**
Age	−0.075	−0.037	−1.124	0.261	−0.206~0.056		
Sex	−0.015	0.000	−0.015	0.988	−2.005~1.975		
Educational level	−1.180	−0.054	−1.597	0.111	−2.630~0.270		
Marital status	0.230	0.021	0.679	0.498	−0.436~0.896		
Income level	0.256	0.016	0.431	0.667	−0.910~1.422		
Number of chronic diseases	−0.438	−0.022	−0.718	0.473	−1.636~0.760		
ADL	0.112	0.081	1.980	0.048	0.001~0.223		
**Block 2. Caregivers’ Demographic Characteristics**						**0.103**	**0.069**
Age	0.030	0.019	0.549	0.583	−0.078~0.139		
Sex	0.594	0.017	0.573	0.567	−1.441~2.629		
Educational level (<senior middle school=0; ≥senior middle school=1)	1.036	0.063	1.641	0.101	−0.203~2.275		
Monthly household income of caregivers	1.685	0.101	2.662	0.008	0.443~2.927		
Marital status (married=1;others=0)	−0.419	−0.026	−0.888	0.375	−1.345~0.507		
Chronic disease (yes=1; no=0)	−3.747	−0.103	−3.428	0.001	−5.892~−1.602		
Relationship to the patient	1.186	0.150	4.556	<0.001	0.675~1.697		
Currently employed (yes=1; no=0)	3.279	0.087	2.454	0.014	0.657~5.901		
Living with the elderly (yes=1; no=0)	−2.294	−0.066	−2.202	0.028	−4.338~−0.249		
**Block 3. Objective Caregiving Loads**						**0.129**	**0.026**
Caregiving in assistance for daily living	−1.130	−0.060	−1.154	0.249	−3.053~0.792		
Caregiving in assistance for housekeeping	−1.051	−0.055	−1.193	0.233	−2.779~0.678		
Caregiving in assistance for transportation	0.967	0.054	1.269	0.205	−0.529~2.464		
Caregiving in assistance for health care	−0.078	−0.004	−0.086	0.931	−1.844~1.689		
Numbers of caregivers	−0.695	−0.097	−3.202	0.001	−1.121~−0.269		
Time spent in caregiving everyday	−0.614	−0.044	−1.238	0.216	−1.587~0.359		
Care duration	−0.007	−0.022	−0.727	0.468	−0.025~0.012		
**Block 4. Subjective Caregiver Burden**	−0.428	−0.364	−12.049	<0.001	−0.497~−0.358	**0.251**	**0.121**
**Block5. SOC**	0.227	0.072	2.526	0.012	0.051~0.403	**0.255**	**0.004**

**Table 4:** Hierarchical multiple regression predicting the MCS scores

	**Estimate (B)**	**Standardized estimate (β)**	**t**	***P***	**95% CI of β**	**R**^**2**^	**△R**^**2**^
Constant	45.489		5.713	<0.001	29.863~61.115		
**Block 1. The Elderly’ Characteristics**						0.031	0.031
Age	−0.015	−0.007	−0.231	0.817	−0.141~0.111		
Sex	1.449	0.042	1.488	0.137	−0.462~3.360		
Educational level	−0.507	−0.024	−0.715	0.475	−1.901~0.886		
Marital status	−0.008	−0.001	−0.024	0.981	−0.648~0.633		
Income	0.294	0.018	0.515	0.607	−0.827~1.415		
Number of chronic disease	−0.196	−0.010	−0.333	0.740	−1.350~0.958		
ADL	0.102	0.075	1.851	0.065	−0.006~0.210		
**Block 2.Caregivers’ Demographic Characteristics**						0.072	0.041
Age	0.130	0.085	2.440	0.015	0.025~0.234		
Sex	−0.865	−0.025	−0.870	0.385	−2.817~1.087		
Educational level (<senior middle school=0; ≥senior middle school=1)	0.851	0.053	1.403	0.161	−0.339~2.040		
Monthly household income	1.214	0.074	2.004	0.045	0.025~2.403		
Marital status(married=1;others=0)	−0.567	−0.035	−1.252	0.211	−1.455~0.321		
Chronic disease (yes=1; no=0)	−2.459	−0.070	−2.107	0.035	−4.749~0.169		
Relationship to the patient	1.027	0.133	4.107	<0.001	0.536~1.517		
Currently employed (yes=1; no=0)	0.960	0.026	0.749	0.454	−1.556~3.476		
Living with the elderly (yes=1; no=0)	−0.753	−0.022	−0.753	0.452	−2.717~1.211		
**Block 3. Objective Caregiving Loads**						0.094	0.022
Caregiving in assistance for daily living	−1.072	−0.058	−1.141	0.254	−2.916~0.772		
Caregiving in assistance for housekeeping	−1.262	−0.068	−1.493	0.136	−2.922~0.397		
Caregiving in assistance for transportation	0.307	0.017	0.421	0.674	−1.126~1.741		
Caregiving in assistance for health care	0.704	0.038	0.814	0.416	−0.993~2.402		
Numbers of caregivers	−0.390	−0.055	−1.866	0.062	−0.800~0.020		
Time spent in caregiving everyday	−0.895	−0.066	−1.884	0.060	−1.828~0.037		
Care duration	0.000	−0.001	−0.047	0.963	−0.018~0.017		
**Block 4. Subjective Caregiver Burden**	−0.490	−0.427	−14.388	<0.001	−0.557~−0.423	0.266	0.173
**Block5. SOC**	0.397	0.129	4.597	<0.001	0.227~0.566	0.282	0.016

## Discussion

The results from this study indicate that most Chinese caregivers of the elderly suffer from impaired QOL. This was much lower than the QOL levels of the general mainland Chinese adults, and the Chinese caregivers for the elderly in Hong Kong [24,25]; but higher than the caregivers of stroke patients [26]. And this was consistent with Saunders’ study which found that the caregivers of the heart failure patients felt their health had worsened because of caregiving [27]. Moreover, mental QOL of the caregivers was disrupted more seriously than physical QOL.

In this study, Subjective Caregiver Burden was the strongest predictor of both PCS and MCS. Both physical and mental QOL of caregivers were best predicted by Subjective Caregiver Burden and the Demographic Characteristics of Caregivers. In addition, caregivers’ mental QOL was also predicted by the patients’ characteristics. These findings suggested that not only the objective aspects of caregiving, but also subjective caregiver burden were associated with caregivers’ QOL. Caregiving has disproportionate burdensome effects on all aspects of QOL. Additionally, individual personal coping capability like SOC played a positive role in alleviating burden and could optimize QOL. This is in support with previous studies [23,28] that positive beliefs influence the appraisal of stressful situations and so, could promote health and well being.

In this study we found that the functional dependency of the elderly had significant impacts on the caregivers’ physical health. Our finding is consistent with the existing literature that patients' functional state was significantly related to caregivers' psychosocial burden and was linked to caregiver’s QOL [29,30]. This takes the results of existing literature further in showing that caring for the elderly with low daily living abilities, is associated with caregivers’ poor physical QOL. Caregiving in assistance for daily living, housekeeping, transportation and health care, time spent in caregiving everyday and care duration in the block 3 of objective caregiving loads were not associated with PCS, while the number of caregivers was the predictors of PCS. Caregivers caring for the elderly with high functional dependency engaged in more physical expenditure, which usually led to lack of physical vigor and strength. Therefore, the degree of dependency for assistance of daily living can well predict physical well being of caregivers. Further interventions to improve caregivers’ QOL should target at improving poorer functioning of the elderly.

Currently in China there are no community services or long-term care services for neither the elderly nor chronically ill patients, which makes it difficult for older adults and their families to afford expensive medical treatment and care tasks. Sometimes caregivers had to stop working in order to give full-time care to the elderly. In the present study, we found that nearly 30% of the caregivers only took care of the elderly, they are more likely to spend more time taking care, experiencing physical conflicts of caring tasks, which may be the cause of poor physical health. Lower household income and loss of employment due to caregiving added to caregivers’ susceptibility and vulnerability to perceive the weight of the burden and finally result in decreased physical health. Another reason might be that the employed caregivers could get the benefits of working when giving care to older adults, which was in agreement with previous study [31]. This finding sheds light on the effects of income and employment on caregiver’ QOL.

Caregivers suffering from chronic diseases usually encountered numerous negative repercussions on both physical and mental health. The most likely reason was that their disease put them at a higher risk to lose physical strength and vitality. This subsequently resulted in decreased physical and mental health [23,32,33]. In China, most Chinese caregivers neglected their own health status [33], focusing more, if not completely, on the illness of the elderly. In this frame of mind they did not perceive the impacts of this placed on them, although the physical or mental evidence was present. Hence, the caregivers’ physical and mental well being were decreased due to an increased risk of stress-related mortality.

The caregiver’s relationship with the elderly was strongly related to caregivers’ physical and mental health. In this study, adult children of the elderly accounted for the majority of the family caregiver (66.5%). In China, it is an obligation to provide care for the elderly, owing to the Confucian thought of filial piety and the one-child policy. The adult children of the elderly take upon themselves heavy caring obligations. They have to sacrifice themselves to repay their parents [34], resulting in their physical and mental health being affected greatly.

In this study, we also found that living with the elderly was a significant predictor of physical QOL. The reason may be that, the caregivers living with the elderly, have to manage more stressful and difficult circumstance of caring, as well as spend more time and vigor undertaking the care tasks, which would compromise physical health.

Subjective Caregivers Burden held the most weight in interpretation of QOL, accounting for 12.1% and 17.3% of the observed variability in physical and mental QOL respectively. This is similar to Saunders’ study which shows that Caregiver Burden explained most (62%) of the variance in caregiver’ HRQOL of the older patients with heart failure [35]. Caregivers experiencing heavier burden, reported lower levels of both physical and mental QOL. Assuming the responsibility of caring for the elderly may result in significant disturbances on the caregivers’ time. This makes it difficult to find spare time away from their caregiving obligation to relax or attend social activities and relieve themselves. Caring for an ill family member may actually result in an imbalance between personal affairs and family function. Subjective Caregivers Burden compromises both the physical and mental health status of the caregivers, resulting in an increased risk of stress-related diseases. Caregivers, who view the elderly illness as problematic or consider treatment to be stressful, feel more uncertain and hopeless. They are also much more likely to appraise the caring responsibilities negatively and tend to be emotionally distressed and show physical symptoms. Personal subjective appraisal of the same stressors may be different owing to the caregivers’ abilities to self-regulate and cope. Subjective consciousness always determines psychological discomfort by determining whether the caregiver perceives the burden. As is consistent with other studies, it has been deduced that QOL in caregivers is closely associated with the caregiver’s subjective perception of the impact of caring for the elderly [36,37].

In this study, SOC was moderately and positively associated with both physical and mental health, which was consistent with previous studies [38,39]. It was found that caregivers with high SOC scores enjoyed high physical and mental QOL. Caregivers with a higher SOC, when providing caregiving to the elderly, could choose the adaptive coping response and mobilize resources to handle the difficult caring circumstances. SOC could help the caregivers feel more confident in dealing with the care tasks, improving their abilities to confront stressors. SOC is an orientation to life that can help caregivers avert emotional discomforts in stressful situations and protect both mental and physical health. The caregivers with high SOC consider stressors to be more predictable, and view the stressors as worthwhile and meaningful during the caring process [40].

It is highly recommended that health care resources for the elderly, supporting their caregivers be optimized, especially by delivering psychological counseling services. Targeted support for the most burdened caregivers, such as skills training to aid in alleviating burden and adaptive coping strategies should also be provided.

The present study bears the limitations that it is characterized by cross-sectional research, therefore one cannot derive any conclusions on the causality of the associations observed between caregiving burden and QOL. Moreover, variables related to nurses who could give assistance to caregivers in the communities were not considered because currently in China there are no nurses who assist caregivers in the caregiver role in the home setting. Finally, caregivers were selected by convenience sampling, which may limit the generalizability of this study to other populations. However, despite the above limitations, this study has notable strong points. Firstly, the sample size was quite large. Secondly, there was a high response rate, most likely due to the fact that face to face interviews allowed for more collection of information. Finally, 15 communities in 3 eastern cities (Shenyang, Yantai and Nanqiao of Shanghai) of China were selected. These cities are located at the north, central and south of eastern China. These three cities contain large elderly populations. Thus, this could represent the care situation of most eastern areas of China.

## Conclusions

Most Chinese caregivers of the elderly experienced low levels of HRQOL. Mental QOL were disrupted more seriously than physical QOL. A decrease in caregiver burden ought to promote better management of caregiving tasks, and improve QOL. Also, coping capability, like SOC, are needed to decrease the impact of caregiving on HRQOL of Chinese caregivers.

## Abbreviations

QOL: Quality of Life; HRQOL: Health Related Quality of Life; SF-36: the 36-item Short-Form Health Survey; PCS: Physical Component Summary; MCS: Mental Component Summary; ZBI: the Zarit Caregiver Burden Interview; SOC: Sense of Coherence; HMR: Hierarchical Multiple Regression analysis.

## Competing interests

The authors declare that they have no competing interests.

## Authors' contributions

XY the data collection, statistical analysis and drafting and revision of the manuscript. YH contributed to interpretation and collection of the data, SMG contributed to the data collection and revision of the manuscript. LW was responsible for the development, design. All authors read and approved the final manuscript.
